# Autophagy and Transporter-Based Multi-Drug Resistance

**DOI:** 10.3390/cells1030558

**Published:** 2012-08-23

**Authors:** Priyank Kumar, Dong-Mei Zhang, Kurt Degenhardt, Zhe-Sheng Chen

**Affiliations:** 1 Department of Pharmaceutical Sciences, College of Pharmacy and Allied Health Professionals, St. John’s University, Queens, NY 11439, USA; Email: kpriyank02@gmail.com; 2 College of Pharmacy, Jinan University, Guangzhou 510632, China; Email: dmzhang701@foxmail.com; 3 Department of Basic Sciences, Touro College of Osteopathic Medicine, New York, NY 10027, USA

**Keywords:** autophagy, MDR, apoptosis

## Abstract

All the therapeutic strategies for treating cancers aim at killing the cancer cells via apoptosis (programmed cell death type I). Defective apoptosis endow tumor cells with survival. The cell can respond to such defects with autophagy. Autophagy is a cellular process by which cytoplasmic material is either degraded to maintain homeostasis or recycled for energy and nutrients in starvation. A plethora of evidence has shown that the role of autophagy in tumors is complex. A lot of effort is needed to underline the functional status of autophagy in tumor progression and treatment, and elucidate how to tweak autophagy to treat cancer. Furthermore, during the treatment of cancer, the limitation for the cure rate and survival is the phenomenon of multi drug resistance (MDR). The development of MDR is an intricate process that could be regulated by drug transporters, enzymes, anti-apoptotic genes or DNA repair mechanisms. Reports have shown that autophagy has a dual role in MDR. Furthermore, it has been reported that activation of a death pathway may overcome MDR, thus pointing the importance of other death pathways to regulate tumor cell progression and growth. Therefore, in this review we will discuss the role of autophagy in MDR tumors and a possible link amongst these phenomena.

## 1. Introduction

Biosynthesis and degradation are two processes that regulate the maintenance of internal homeostasis of an organism. Intracellular degradation is governed by two distinct systems: the ubiquitin-proteasome and the lysosome-autophagy system. The ubiquitin-proteasome system targets short-lived and misfolded proteins, while the lysosome-autophagy system additionally can target long‑lived macromolecular complexes and organelles [1]. Autophagy, derived from two Greek words, auto (self) and phagy (eating), coined by Christian de Duve, describes the lysosomal degradation phenomenon. This is a cell’s routine process and strategy that is developed in eukaryotic cells to deal with the stressful stimuli as well as to maintain homeostasis in cells. On the basis of targeted substrates, regulation and selectivity, autophagy is classified as microautophagy, chaperone-mediated autophagy, and macroautophagy [2]. However, the above-mentioned classes of autophagy all have the lysosome as the end point. 

Microautophagy participates in the continuous basal turnover by forming random invaginations in the lysosomal or vacuolar membrane that differentiate into the autophagic tube to enclose the portions of cytosol. In 1981, chaperone-mediated autophagy (CMA) was discovered [1]. In CMA the cytosolic chaperone protein, heat shock cognate 70 (hsc70), targets only single proteins with KEFRQ or KEFRQ-like motif, then binds to LAMP-2A followed by the transfer of chaperone-target protein complex to the lysosomes. Hereafter, macroautophagy will be referred to as autophagy, and is an evolutionarily conserved, genetically controlled process of bulk degradation by which long-lived cellular proteins and organelles are targeted to lysosomes [1,2,3]. In mammals, the basal levels of autophagy might occur to get rid of superfluous, damaged or aged proteins and organelles and genomic instability, to protect cells from external, as well as internal insults [2].

Autophagy is a distinct form of membrane trafficking [4]. It starts from the formation of a crescent shaped isolation membrane (phagophore) that extends and sequesters cytoplasmic organelles and macromolecules, which is then called pre-autophagosomal structure (PAS). When PAS creates a complete closed double membranous structure, it is called the autophagosome. The autophagosome then undergoes a series of maturation events and fuses with lysosomes, thereby giving life to the autolysosome and degrading the material contained in it by the help of acid hydrolases. In some cases, autophagosomes may also fuse with endosomes, which then are called amphisomes. The digestion of sequestered material in the lysosomes forms amino acids, fatty acids and nucleotides, which are recycled to generate ATP and macromolecular synthesis [2,5,6]. Autophagy defects make mice more vulnerable to genomic instability, metabolic damage and tumorigenesis, indicating a role of autophagy in tumor suppression [6]. Monoallelic loss of the essential autophagy gene, *Beclin1*, has been reported in 40%–75% of human breast, prostate and ovarian cancers, pointing at the probable role of autophagy in preventing these tumors [7]. However, autophagy also provides tumor cells with strength to fight stress caused by high metabolic demand associated with an increased rate of cell proliferation [6]. Autophagy is induced in hypoxic regions of tumor cells [8]. In preclinical studies, repression of autophagy has demonstrated an increased efficacy of chemotherapeutics, both *in vitro,* as well as *in vivo* [6,9]. There are a number of reports stating the importance of autophagy in tumor cell metabolism [10,11,12,13,14].

One of the most unnerving clinical issues is the recurrent tumor progression after treatment, due to resistance to structurally and mechanistically unrelated compounds [15]. This phenomenon of resistance is called multidrug resistance (MDR). Therefore, the development of novel anticancer agents with low toxicity and overcoming the MDR phenotype are the major goals of oncology research. Various mechanisms have been described to explain the phenomenon of MDR in mammalian cells. Often in MDR, the antineoplastic agents are extruded from the tumor cells, hence, reducing the intercellular concentration of antineoplastic agents below the cytotoxic threshold [16]. 

## 2. Mechanisms Regulating MDR

**Figure 1:**
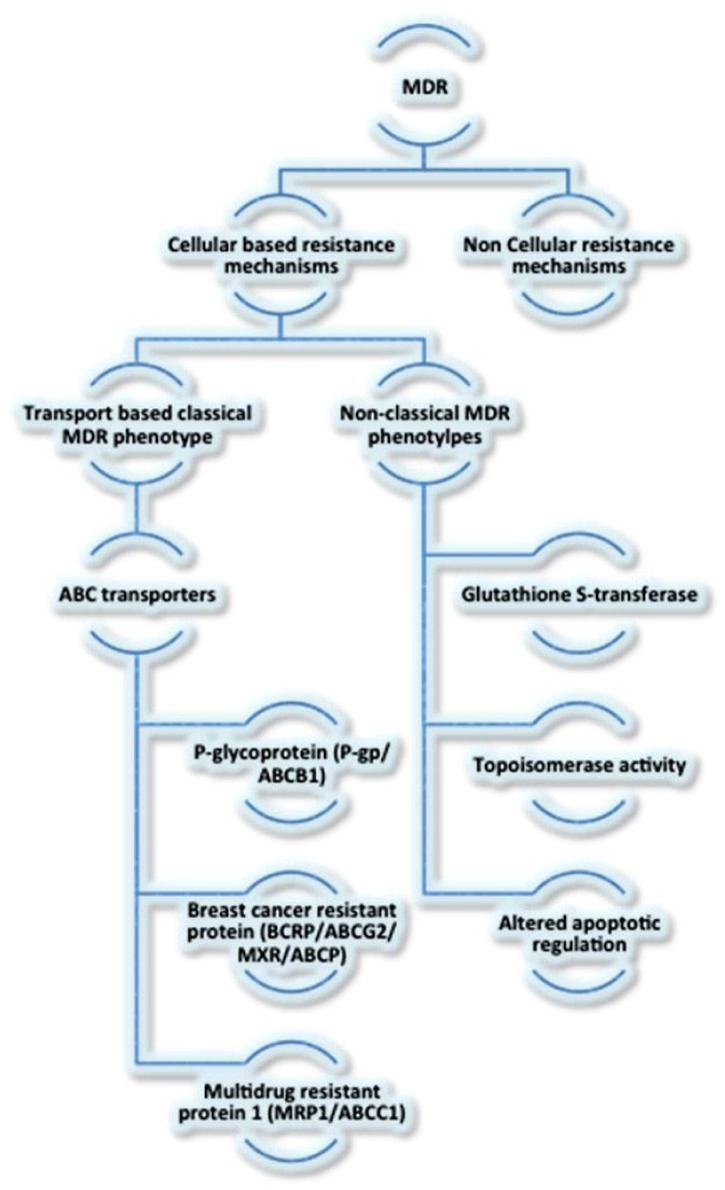
Classification of MDR. A flowchart illustrating the classification of MDR.

On the basis of mechanisms of resistance, MDR is broadly classified as non-cellular and cellular mechanisms as discussed below (Figure 1) [17].

### 2.1. Non-Cellular Mechanisms

The phenomenon of non-cellular drug mechanisms is associated with some types of solid tumors. Poor tumor vascularization and ischemic regions in solid tumors can induce resistance to chemotherapeutic agents. The acidic pH in ischemic regions of the tumor microenvironment is due to lactic acid generation, which may also contribute to the resistance to weak bases [18].

### 2.2. Cellular Mechanisms

Cellular-based resistant mechanisms are further classified into non-classical MDR phenotypes and transport-based classical MDR phenotype. Non-classical MDR is acquired by the tumors because of the activity of some specific enzymes, such as glutathione S-transferase and topoisomerase [17]. Moreover, changes in proteins regulating apoptosis can also lead to a reduction of sensitivity to certain anticancer drugs, since most of these drugs kill tumor cells via apoptosis. On the other hand, the ATP binding cassette (ABC) family of transporters is involved in transport-based classical MDR. Hereafter, in this review, classical transport-based MDR will be referred to as MDR [17]. MDR studies suggested that drug transport is a process that is carefully regulated and controlled by members of the ABC transporter family of proteins [15]. So far, 49 different types of human ABC transporters are identified. On the basis of sequence similarities, as well as structural organization, the ABC transporters are classified into seven subfamilies from ABC-A to ABC-G. Sub family C is composed of 13 members, nine of which are called multidrug resistant proteins (MRPs) [15,19]. Several reports have shown that three ABC transporters cause most human MDR: P-glycoprotein (P-gp/ABCB1), multidrug resistant protein 1 (MRP1/ABCC1) and breast cancer resistant protein (BCRP/ABCG2/MXR/ABCP). Several evidences show that inhibition of these above-mentioned transporters can reverse MDR [15,19]. However, complete remission of MDR is not seen even after the combination treatment of inhibitor and chemotherapeutic agent, indicating that there may be an intervention of some other pathways in MDR. In this review, we will discuss the interaction of Autophagy and MDR.

## 3. Regulation of Autophagy

Although autophagy occurs as a continuum, it is conceptually helpful to dissect the core autophagic pathway into several key stages. These stages include initiation and formation of the phagophore, autophagosome formation and lysosomal fusion. This core pathway has been the focus of several review articles that thoroughly discuss the roles of many proteins, as well as several lipids [20,21] that are involved in each of the stages. Here, we will briefly discuss the core pathway, but focus on some exciting pathways that regulate the induction of autophagy, including regulation by HMGB1 and p53.

### 3.1. Phagophore Formation

Cellular survival during metabolic stress depends on the proper implementation of autophagy. The formation of an autophagic vacuole from intracellular membranes that surround the proper cytosolic constituents is complex and requires the interplay of many molecular machines. Although we are starting to understand the mechanism of this process, great progress has been achieved identifying many of the proteins involved in autophagy. A central regulator for the initiation of autophagy is Beclin1 (Bcl-2 interacting coiled-coil protein 1; also known as ATG6) [22]. In fact, Beclin1 heterozygote mice have both a severe autophagic defect and a tumor-prone phenotype [23].

An early observation that linked the regulation of autophagy and apoptosis is in non-stressed cells, where Beclin1 forms a complex with, and is inhibited by, Bcl2, a central anti-apoptosis regulator [22]. Commitment to autophagy coincides with the release of Beclin1 and the formation of a complex, including UVRAG, p150 (a myristoylated PI3kinase anchoring protein) and PI3Kinase (class III not to be confused with insulin receptor activated PI3K class I). This protein complex phosphorylates phosphotidylinositol (PI), generating PI triphosphate (PtdIns(3)P). Formation of PI triphosphate coincides with the expansion of the isolation membrane [24,25,26,27,28,29,30]. 

### 3.2. Autophagosome

Formation of the autophagosome from the phagophore requires recruitment of Atg 12, Atg5, Atg 7, Atg10, Atg16, and the subsequent post-translational lipidation of LC3 (microtubule associated protein 1 light chain3/Atg8). Lipidation of LC3-I requires post-translational conjugation of Atg12 with Atg5 (this process is analogous to ubiquitin conjugation) [31,32,33]. Following recruitment to the phagophore, Atg12 is catalytically processed (similar to ubiquitin activation) by Atg7 (an E1 ubiquitin-like enzyme) in an ATP-dependent manner [34]. The modified Atg12 is conjugated to Atg5 by Atg10 (an E2 ubiquitin-like enzyme). The newly formed Atg12-5 conjugate (similar to an E3 ubiquitin-like enzyme) forms a complex with Atg16 and functions with Atg7 (an E1) and Atg3 (an E2) to conjugate LC3-1 with phosphatidylethanolamine (PE) [35,36,37,38,39].

Interestingly, LC3 undergoes a series of post-translation modifications prior to joining the phagophore. First, the c-terminal arginine of nascent LC3 is cleaved off by the cysteine protease Atg4 to form LC3-1. The c-terminal amino acid of cytosolic LC3-1 is a glycine. Now, LC3-1 can be lipidated by the Atg7, Atg3, and the Atg12-5 conjugate to form LC3-II [40,41,42,43]. LC3-II remains with the autophagosome; however, the Atg12-5 conjugate dissociates once the autophagosome is formed.

#### Fusion of the Autophagosome with the Lysosome

Although it has long been known that autophagosomes with cytosolic contents fuse with lysosomes for degradation, the molecular mechanism underlying the process is largely unknown. Recently it has been reported that TECPR1 (Tectonin domain-containing protein 1) binds to the Atg12-Atg5 conjugate and phosphatidylinositol 3-phosphate (PtdIns(3)P), and this interaction promotes autophagosome-lysosome fusion [44]. 

### 3.3. Regulation

#### 3.3.1. Beclin1: Promiscuous Binding Regulates Autophagy

**Figure 2:**
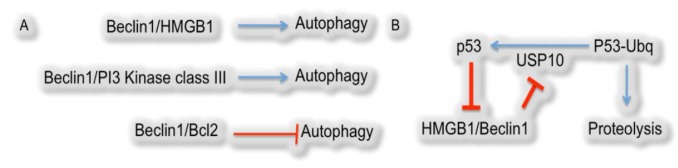
Summary of regulatory mechanisms of autophagy. (**A**) Beclin1 binding to HMGB1, as well as PI3 Kinase class III, positively regulates autophagy. However, Beclin1 binding to Bcl2 negatively regulates autophagy. (**B**) p53-mediated downregulation of autophagy.

Beclin1 has multiple binding partners and it seems the binding partner determines Beclin1-mediated alterations in cellular physiology. As described above, Beclin1 associates with PI3Kinase class III to promote autophagy, while Beclin1-mediated autophagy is inhibited by complex formation with Bcl2. Cellular physiology is under the influence of another recently identified heterodimer combination, Beclin1/HMGB1 (Figure 2A) [20,45].

#### 3.3.2. HMGB1 Binding to Beclin1 Promotes Autophagy

The chromatin binding protein high mobility group box 1 (HMGB1) has a well-characterized damage-associated molecular pattern. It is found in the nucleus, bound to chromatin, in undamaged cells. HMGB1 is released from the nucleus and has a cytosolic localization in damaged but living cells while it is released from necrotic cells into an extracellular environment [46]. This versatile molecule is even released from macrophages as an inflammatory regulatory molecule [47,48,49]. During metabolic stress, HMGB1 translocates from the nucleus to the cytosol. The cytosolic localization promotes Beclin1 complex formation and induction of autophagy potentially by disrupting Bcl2 from binding to and inhibiting Beclin1-mediated autophagy (Figure 2A) [46].

#### 3.3.3. p53 Determines Cell Fate: Apoptosis or Autophagy

Autophagy cannot escape control by p53. p53, the prototypical tumor suppressor, regulates autophagy-mediated homeostasis, by monitoring the rate of autophagy to the varying nutrient availability [50]. Cytosolic p53 complexes with HMGB1; this precludes HMGB1 from promoting Beclin1-mediated autophagy [51]. So, p53, in addition to inducing apoptosis and cell-cycle arrest, can down-regulate autophagy. However, Beclin1 indirectly inhibits p53. Beclin1 controls the protein stability of USP10. USP10 is a de-ubiquitinating enzyme that promotes accumulation of p53, so Beclin1 mediates down-regulation of USP10 and reduces p53 levels [52]. Therefore, it leads to either induction of apoptosis and inhibition of autophagy by p53 or induction of autophagy and inhibition of p53 by Beclin1 (Figure 2B) [51].

#### 3.3.4. Negative Regulation of Autophagy by the PI3Kinase/AKT/mTOR Pathway

**Figure 3:**
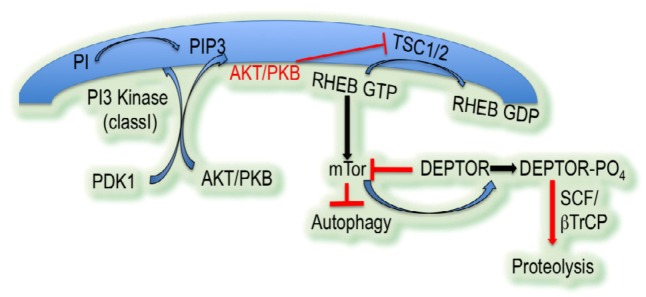
Negative regulation of autophagy by PI3Kinase/AKT/mTOR pathways followed by DEPTOR-mediated positive feedback loop for mTOR.

Activation of receptor tyrosine kinases (RTK) inhibits autophagy. RTK activation promotes activation of Ras (rat sarcoma) signaling, including activation of PI3Kinase class I. PI3Kinase class I phosphorylates ptdIns(4,5)P2 to form PtdIns(3,4,5)P3 at the plasma membrane. This molecule signals recruitment of AKT (also known as protein kinase B) and phosphoinositide-dependent protein kinase1. Co-recruitment of these two kinases leads to activation of AKT. Active AKT promotes accumulation of RHEB GTP by phosphorylating and inhibiting TSC1/TSC2, RHEB GTPase activating proteins. RHEB GTP activates mTOR (mammalian target of rapamycin). Activation of this serine/threonine kinase is a potent inhibitor of autophagy (Figure 3) [20].

AKT activation and inhibition of autophagy is predicated on the accumulation of PtdIns(3,4,5)P3. PTEN, a potent tumor suppressor that is deleted in many cancers, is a phosphatase that converts PtdIns(3,4,5)P3 to PtdIns(4,5)P2. This prevents activation of AKT, mTOR, and the subsequent inhibition of autophagy [20].

#### 3.3.5. DEPTOR: A Positive Feedback Loop for mTOR

DEPTOR, a DEP domain containing mTOR-interacting protein, is an endogenous inhibitor of mTOR. DEPTOR is degraded by the 26S proteasome in response to phosphorylation by mTOR leading to SCF (TrCP)-mediated degradation. Thus activation of mTOR leads to reduced inhibition of DEPTOR and a positive feedback loop controlling autophagy [53]. Thus, inhibition of autophagy by mTOR is exacerbated by the decreased affect of DEPTOR (Figure 3).

## 4. Autophagy: Savior or Satan for Tumor Cells?

Extensive studies indicate that autophagy plays a controversial role in the survival and death of cancer cells [54,55,56]. In general, autophagy confers a prosurvival mechanism that protects cancer cells from various stresses, such as amino acid deprivation, hypoxia, DNA and mitochondria damage, oxidative stress, *etc.* Autophagy is activated when the cells respond to the limited nutrition and growth factors and contributes to maintaining homeostasis through degradation of impaired or unnecessary macromolecules and organelles, thereby providing energy to cancer cells [5,57,58]. Increased basal level of autophagy was observed in some human tumor tissues. A positive correlation between poor patient outcome and enhanced autophagy level in cancer patients suggests that the occurrence of autophagy may be associated with increased cancer progression [59,60]. Recently, some molecular mechanisms underlying the cytoprotective role of autophagy have been revealed [61]. The E3 ubiquitin ligase c-Cbl, as an autophagosome cargo receptor for Src, targets active Src to promote autophagic degradation and ensures tumor cell survival [62]. It is well known that the energy sensor AMP-activated protein kinase (AMPK) triggers autophagy through inactivation of mTOR complex-1 (mTORC1), while direct phosphorylation of ULK1 at several different sites was also found to be involved in the AMPK-induced autophagy [63,64]. BAX inhibitor-1 deficiency leads to autophagy to improve cell growth under starvation conditions by controlling the IRE1α branch of the unfolded protein response [65]. *H*-*Ras* or *K*-*Ras* oncogene mutation up-regulates basal autophagy, which is required for tumor cell survival in starvation and in tumorigenesis [66]. Highly expressed receptor for advanced glycation end products (RAGE), as an inflammatory receptor, contributes to the activation of interleukin 6 (IL-6)-mediated mitochondrial pathway and transcription 3 (STAT3) signaling, which activates autophagy, inhibits apoptosis, thereby promoting cell survival in pancreatic cancer *in vivo* and *in vitro* [67,68]. It is also found that lymphocyte-induced cell-mediated autophagy promotes tumorigenesis, which hints that autophagy may be an important link between inflammation and tumorigenesis [10,11,12]. On the other hand, autophagy is also considered to contribute to tumor recurrence and progression after radiation therapy and chemotherapy [69]. For example, suberoylanilide hydroxamic acid, a histone deacetylase inhibitor, can induce autophagy via inhibiting the mTOR pathway which antagonizes apoptosis [70]. Therefore, inhibition of prosurvival autophagy by genetic or pharmacological methods might improve the efficacy of chemotherapy, radiotherapy, and immunotherapy [71]. Combination of chloroquine (CQ), hydroxychloroquine or 3‑methyladenine (3-MA) with anticancer drugs leads to increased cytotoxicity in preclinical and clinical models [69,72]. CQ-inhibiting autophagy during Interleukin 2 immunotherapy promotes long term tumor regression [73].

In contrast to the well-documented cancer-promoting effect of autophagy, a function of autophagy in an anticancer role has been proposed. Accordingly, some oncogenic proteins (Bcl-2, PI3K, Akt1, *etc.*) inhibit autophagy, while some tumor suppressor proteins (like Beclin1, Bif-1, LKB1, UVRAG) trigger autophagy [13,56,74]. For example, Beclin1, which forms an autophagic core complex with Vps34 and Vps15, is monoallelically lost in human ovarian, breast, and prostate tumors. Moreover, Beclin1^+/−^ mice are tumor-prone, but its high expression inhibits the growth of human cancer cells [75]. The mechanisms by which autophagy inhibits tumor development have been put forward. It has been found that autophagy reduces mutagenesis, oncogene activation, or tumorigenesis, by removal of damaged mitochondria and other organelles that are caused by accumulation of p62, reactive oxygen species (ROS), and protein aggregation, thereby inhibiting the cancer cell growth in the early stage of cancer development [54,74]. It has been well proven that autophagy inhibition leads to tumorigenesis by elimination of p62/SQSTM1 [76,77]. Autophagy also suppresses tumors by cooperating with apoptosis to cause cell death [78]. Autophagy occurs as a primary response to stress stimuli and subsequently triggers either apoptosis or necrosis to eliminate cancer cells. In this scenario, autophagy as a partner or an enabler of apoptosis is necessary for apoptosis induction [79]. It has been found that caffeine induces apoptosis by enhancement of autophagy via PI3K/Akt/mTOR/p70S6K signaling pathway inhibition. Blockade of autophagy, by either 3-MA or siRNAs specific for *Atg7* genes, could partially attenuate the apoptotic process [80]. Similar phenomena were also observed in the progress of oleostearic acid or YM155-induced cancer cell death [81,82]. Furthermore, autophagy also inhibits tumors by autophagic cell death (type II programmed cell death) [78]. Autophagic cell death is presumed to result from excessive levels of cellular autophagy, causing irreversible damage to cells through selective degradation of regulatory molecules or organelles, such as mitochondria, endoplasmic reticulum, and golgi that are essential for cell survival [83]. Several proteins have been indicated to be involved in autophagic cell death, such as Ras, E4F1, Foxo1, histone deacetylases, steroid receptor coactivator 3, *etc.* [84,85,86,87,88]. Intensive studies report that many compounds with diverse structures can induce autophagic puncta and increase autophagic flux [89]. For example, dasatinib (an inhibitor of Src/Abl family kinases) induces autophagic cell death in the human ovarian cancer xenograft model. Small hairpin RNA knockdown of Beclin1 expression reduced dasatinib-induced autophagy and growth inhibition [14]. However, some doubts on the actual existence of autophagic cell death were proposed recently [89,90]. The notion that autophagy is a real killer, an accomplice or just an innocent by-stander in the progress of cell death still needs to be further studied. 

Taken together, the double face of autophagy in tumor cell survival and suppression is complex and context-dependent. A better understanding of molecular mechanisms of autophagy, as well as the role of autophagy, in different stages of carcinogenesis will help develop new approaches for the prevention and therapy of tumors. 

## 5. How Does Autophagy Communicate with MDR?

The overexpression of ABC transporters is predominantly correlated with drug resistance. Hence, compounds that can inhibit ABC transporter-mediated efflux are developed as potential chemotherapeutic agents against MDR. However, modulators of MDR are not as effective as expected because, besides ABC transporters, various other mechanisms contribute to drug resistance. Recent studies have suggested approaches to exploit autophagy to overcome MDR during anticancer therapy [91]. Results from a report by Meschini *et al.*, confirmed that vocamine, a bisindolic alkaloid from *Peschiera fuchsiaefolia* in combination with doxorubicin, could not only overcome the resistance of resistant osteosarcoma cells by competitively inhibiting P-glycoprotein (P-gp)/ABCB1, a 170 KD protein encoded by *MDR1* gene, but also lead them to autophagic cell death [16]. Kim *et al.*, reported that induction of autophagy in apoptotic deficient H460 lung cancer cells enhances the efficacy of radiation therapy *in vitro*, as well as in the lung cancer xenograft model [92], which suggests the potential of autophagy to overcome MDR. Peculiarly, mTOR, a negative regulator of autophagy, is over-active in most types of cancer [93,94], giving rise to several clinical trials involving inhibitors of mTOR in combination with anticancer agents to achieve better clinical outcomes [95]. A recent report showed that a combination of Beclin1 expression and inhibition of mTOR by rapamycin acts synergistically against the growth of v-Ha-Ras transformed NIH 3T3 (Ras-NIH 3T3/MDR) cells, leading to pronounced inhibition through the induction of autophagy [96]. Furthermore, Mazzanti *et al.*, demonstrated that constitutive expression of P-gp in hepatocellular cancer cells is linked to the HGF/MET autocrine loop, which then leads to overexpression of Bcl2 and mTOR, and inhibition of eIf2α, conferring on these cells resistance to autophagy and apoptosis [97]. Recently, a study was published, stating that autophagy leads to cell death in a P-gp overexpressing paclitaxel resistant breast cancer line (MCF‑7/TaxR) with blocked apoptotic pathway [98], thus suggesting the existence of a switch from apoptosis to autophagy that leads to cell death. Cisplatin-based chemotherapy is widely used for the treatment of many solid tumors; however, the chronic exposure to cisplatin leads to a decreased therapeutic efficacy because of the acquired MDR. Cisplatin-based MDR is attributed to increased levels of metallothionine and glutathione, or enzymes involved in the metabolism of glutathione, P-gp mediated inhibition of cisplatin-induced caspase3 induction [99,100]. Sirichanchuen *et al.*, reported that chronic exposure to barely toxic doses of cisplatin to H460 (H460/cis) cells inhibited autophagy that was shown by a decreased LC3II/LC3I ratio and overexpression of Bcl2 protein, a negative regulator of autophagy. Furthermore, in the same report it was reported that the treatment with TFP, an autophagy inducer, could reverse the resistance of H460/cis cells towards cisplatin-induced apoptosis. This finding indicates that cisplatin-based MDR is also related to the inhibition of autophagy [101]. 

**Figure 4:**
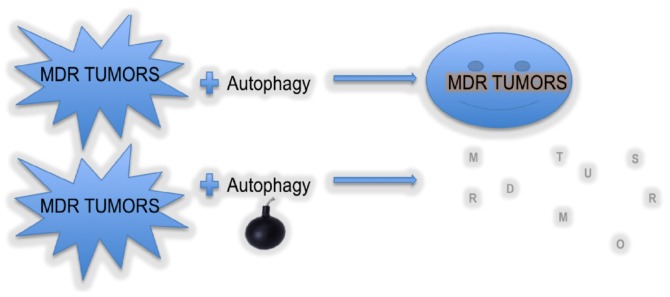
Role of autophagy in MDR tumors. Autophagy has a dual role in MDR tumors. Autophagy may lead to survival of MDR tumors or its activation may lead to tumor cell death.

There is a large body of evidence demonstrating that autophagy is required to kill the MDR tumor cells. However, a recent study showed that the inhibition of autophagy is required to sensitize NIH 3T3/MDR cells to a Src tyrosine kinase inhibitor, PP2 [102], indicating that autophagic blockade in combination with Src tyrosine kinase inhibitor can be a potential therapeutic approach against MDR tumors. The drug of choice for the treatment of colon cancer patients is 5-fluorouracil (5-FU), an antimetabolite, a pyrimidine analog that inhibits the thymidylate synthetase or incorporates itself into nucleic acid, ultimately activating the apoptotic events. However, repeated administration of 5-FU causes MDR, therefore many attempts have been made to increase the therapeutic efficacy of 5-FU. Recently, many reports have reported that autophagy inhibitors can increase the sensitivity of colon cancer cells for 5-FU. For example, 3-MA, an autophagy inhibitor, enhances the 5-FU-mediated apoptosis in colon cancer cells *in vitro* and *in vivo* [103,104]. Similarly, Sasaki *et al.*, showed that combination treatment with chloroquine, a lysosomotropic, can potentiate the 5-FU mediated cell cycle arrest of colon cancer cells to G0/G1 phase by inhibiting the autophagy [105]. Furthermore, it was also shown in a recent report that combination treatment of 5-FU and chloroquine increased the expression of pro-apoptotic proteins, namely Bad and Bax in an *in vivo* colon cancer model, suggesting that this combination treatment can turn out to be a promising strategy to treat colon cancer [106]. It is widely known that p53, a tumor suppressor, is activated in response to insults and leads to cell death or senescence. Mutations in p53 are linked to the failure of chemotherapy or radiotherapy. A recent report demonstrated that both wild type, as well as mutated p53, could reverse the MDR in MDR phenotype of ovarian cancer cells. Interestingly, it was also reported that MDR phenotype ovarian cancer cells use autophagy as a protective phenomenon. However, the different forms of p53 have different mechanisms of action; mutant p53 utilizes autophagy to kill the MDR positive ovarian cancer cells, while wild type p53 inhibits autophagy and reverses the MDR [107]. Thus as per accumulated evidence, we can summarize that autophagy is considered to possess a dual role in cancer development and progression of tumor cell survival, as well as induction of death (Figure 4). The varied nature of autophagy, to a great extent, depends on the type of tumor, stage of disease, and nature of treatment [108].

## 6. Conclusion

Cancer progression is governed by numerous genetic factors; MDR is also regulated by a combination of several drug resistant factors. MDR is an intricate, dynamic and intangible phenomenon. Although, conventional anticancer drugs have multiple targets, MDR seems to be highly unavoidable because anticancer drugs may be substrates and inducers of ABC transporters. Thus, it has been followed as a rule in clinical trials that a modulator of the activity of the ABC transporter in combination with standard anticancer drug is administered, to make resistant cancers responsive to the therapy. However, even this combination treatment does not completely abolish resistance. This makes it important to further elucidate the role of other pathways that may be communicating with MDR. As known, autophagy in healthy cells acts as a janitor by removing the damaged organelles and proteins, reducing the reactive oxygen species, and suppressing necrosis-induced inflammation that can induce cancer when chronic. However, it behaves as a cytoprotectant in established tumors and hence, its inhibition could enhance apoptosis [6]. Although the mechanism of dual interaction of autophagy and MDR is currently unknown, data from recent reports [10,11,12,13,50,75,103,104,105] makes it interesting to investigate this avenue in order to get better clinical outcomes against resistant cancers.
